# Conditional Survival of Advanced Epithelial Ovarian Cancer: A Real-World Data Retrospective Cohort Study From the SEER Database

**DOI:** 10.3389/fmed.2021.756401

**Published:** 2021-11-23

**Authors:** Peng Zheng, Ping Zheng, Guilin Chen

**Affiliations:** ^1^Department of Intensive Care Unit, Fujian Medical University Cancer Hospital, Fujian Cancer Hospital, Fuzhou, China; ^2^Department of Osteology, Fuzhou Second Hospital, Xiamen University, Fuzhou, China; ^3^Department of Gynecological Oncology, Fujian Medical University Cancer Hospital, Fujian Cancer Hospital, Fuzhou, China

**Keywords:** conditional survival, epithelial ovarian cancer, FIGO stage III and IV, prognosis, SEER database

## Abstract

**Objective:** To analyze conditional survival (CS) in patients with advanced epithelial ovarian cancer (EOC) and investigate prognostic factors that affect the CS rate to provide more accurate survival information.

**Methods:** Patients with advanced EOC between 2004 and 2015 were identified from the Surveillance, Epidemiology, and End Results (SEER) database. CS analysis was performed to depict exact survival for patients who had already survived a specific number of years. Cox proportional hazards regression was performed to ascertain the individual contribution of factors associated with actuarial overall survival (OS) at diagnosis and CS at 1, 3, and 5 years after diagnosis.

**Results:** Of 11,773 patients, OS decreased from 32.2% at 6 years to 25.1% at 8 years, while the corresponding 5 year CS (CS5) increased from 37.5% at 1 year to 43.9% at 3 years. Subgroup analysis stratified by clinicopathological characteristics showed that CS5 was always higher than the corresponding actuarial survival (all Δ > 0). Based on multivariate analysis at diagnosis, age, race, marital status, histological type, tumor grade, size, T stage, M stage, surgery, radiation therapy, and chemotherapy were independent prognostic factors for OS. Five years after diagnosis, however, only age, histological type, tumor grade, and laterality were persistently significant independent prognostic factors (all *P* <0.05). Furthermore, patients with poor pathological prognostic factors achieved greater improvements in CS5 rates, and the survival gaps between OS and CS were more obvious.

**Conclusion:** CS of advanced EOC was dynamic and increased over time. Age, histology, tumor grade, and laterality were significant prognostic factors even 5 years after diagnosis. Thus, the availability of updated prognoses at various time points will allow clinicians to better guide their patients.

## Introduction

Ovarian cancer is one of the most common tumors in women, with the highest mortality rate among gynecology malignancies (1, 2). Epithelial ovarian cancer (EOC) is the most common histological type of ovarian cancer, accounting for approximately 90% of cases (3). Primary cytoreductive surgery (PCS) followed by platinum-based chemotherapy has been the standard treatment approach in advanced EOC (4). Although the survival of ovarian cancer has improved over the decades, the prognosis of advanced ovarian cancer remains poor. The 5 year overall survival (OS) rates of patients with Federation International of Gynecology and Obstetrics (FIGO) stage III and IV EOC are 42 and 26%, respectively (3).

The prognosis of patients with cancer mainly depends on stage and the individual's response to treatment, but prognosis can also change for each individual over time. Furthermore, with increased survival, there is also increasing interest in quality of life for advanced epithelial ovarian cancer survivors and their survivorship care. However, traditional Kaplan–Meier assessment can only be used to determine survival and prognosis at the time of diagnosis and does not change with passage of time (5, 6), which fails to reflect the dynamic prognosis updated to the current status.

Recently, conditional survival (CS) assessment has become an accurate and informative assessment method to better predict survival time in patients with cancer (7). CS represents the probability of surviving a certain number of years after diagnosis or treatment based on the time the patient has already survived. This might therefore be more meaningful for patients than conventional survival analysis, as it provides a more individualized prognosis as time progresses. It can also be used by doctors to develop more appropriate treatment regimens and surveillance models. In multiple tumors, such as esophageal, colorectal, lung and breast cancer, previous studies have shown that conditional survival improves over time (7–10).

To the best of our knowledge, no study has specifically examined the conditional survival of patients with advanced EOC. Thus, in this study, we used the Surveillance, Epidemiology and End Results (SEER) database and performed conditional survival analysis to predict more accurate survival for patients with advanced EOC. In addition, changes over time in the prognostic significance of clinicopathological and treatment-related factors were also analyzed.

## Materials and Methods

### Study Population and Characteristics

The data were obtained from the SEER registry of the National Cancer Institute (https://seer.cancer.gov/seerstat/). The SEER database is a national population-based cancer registry that is globally recognized for its accuracy and completeness. Information about cancer incidence and survival for approximately 26% of American people is collected and published by this institute. Since the data from the SEER registry are deidentified and publicly available, no IRB approval was necessary.

**Figure 1:**
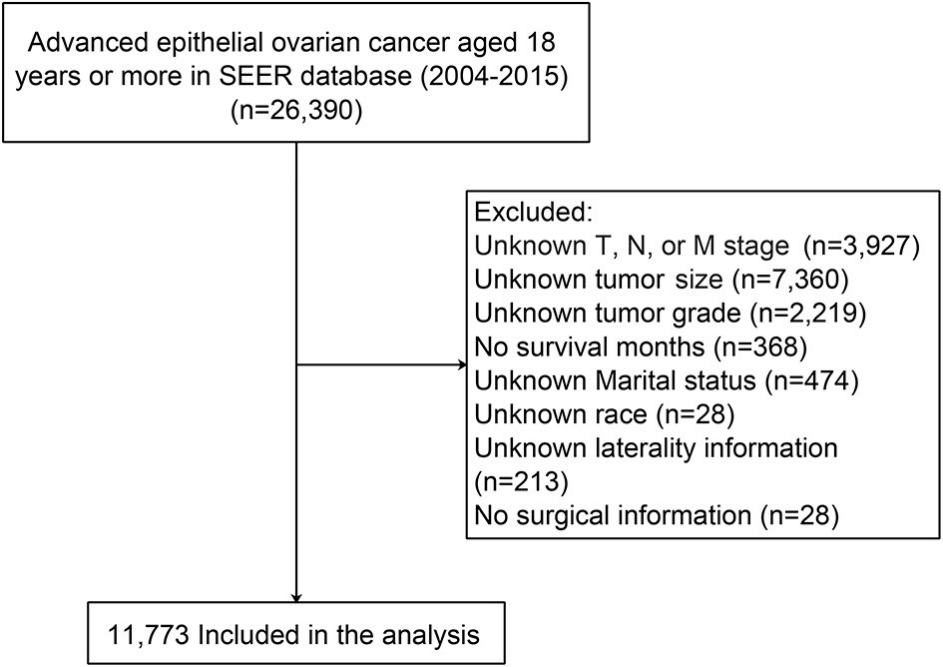
Data selection flowchart.

Patient data were extracted from the latest version of the SEER database using SEER^*^Stat (version 8.3.8) software (Reference number: 10579-Nov2019). The inclusion criteria were as follows: (1) patients were pathologically diagnosed with advanced EOC between 2004 and 2015 (FIGO stage III and IV); (2) patients were at least 18 years of age; (3) ovarian cancer was the only primary carcinoma; and (4) histological code was in accordance with the International Classification of Tumor Diseases, Third Edition (ICD-O-3) (11). Patients with incomplete or unknown clinicopathological information, diagnosed by autopsy only and unknown survival status were all excluded. Finally, information from 11,773 ovarian cancer patients was used in this study (Figure 1).

### Clinical Variables

In this study, clinicopathological features included age, race, marital status, tumor size, laterality, grade, histological type, T stage, N stage, M stage, surgical information, radiotherapy information, and chemotherapy information. In addition, TNM status in this study was redefined according to the American Joint Committee on Cancer seventh edition (AJCC). The optimal cutoff point of tumor size was determined by the median value.

### Statistical Analysis

Continuous variables are expressed as the means and standard deviation (SD), and categorical variables are expressed as numbers. OS was calculated from the day of diagnosis until the day of death from any cause (event) or the last day of follow-up (censored). Survival rates and median overall survival were calculated using Kaplan-Meier analysis. The log-rank test was performed for comparisons between groups.

CS is defined as the probability of surviving an additional number of y years given that a patient has already survived for x years and can be calculated from Kaplan-Meier survival data. For instance, an additional 5-year survival (CS5) was calculated as CS5 = S(x +5)/S(x), which means that CS5 among patients who survived 3 years from the date of diagnosis was calculated by dividing the survival at 8 years by the survival at 3 years. Multivariate Cox proportional hazards regression was performed to evaluate the hazard of the CSS rate at the time of diagnosis and CS rates for multiple survival periods (1, 3, and 5 years after diagnosis) (12). All tests were two-sided, and statistical significance was inferred at *P* <0.050. Statistical analyses were performed using SPSS® version 22.0 (IBM, Armonk, New York, USA) and R version 3.3.3 (R Foundation for Statistical Computing, Vienna, Austria).

## Results

### Demographic and Clinicopathological Characteristics

In total, 11,773 patients were included in our cohort. The median age of these patients was 61 (IQR: 52–69). Most patients were white (84.8%) and married (57.5%). Most subjects were T3 stage (91.0%), grade III & IV (85%), serous histologic type (76.4%), and bilateral (57.8%). There were 4,675 (39.7%) patients with lymph node metastasis and 3,252 (27.6%) patients with distant metastasis. Regarding treatment schemes, 11,608 (98.6%) patients underwent surgery, 10,121 (86.0%) patients received chemotherapy, and 157 (1.3%) patients underwent radiation during their treatment courses (Table 1).

**Table 1:** Baseline clinicopathologic characteristics.

**Characteristics**	***N***	**%**
**Age, y**		
<45	1,081	9.2%
45–64	6,180	52.5%
≥65	4,512	38.3%
**Race**		
White	9,982	84.8%
Black	767	6.5%
Other	1,024	8.7%
**Marital status**		
Single	2,056	17.5%
Married	6,770	57.5%
Divorced or Separated	1,434	12.2%
Widowed	1,513	12.9%
**Histological type**		
Serous	8,995	76.4%
Endometrioid	588	5.0%
Mucinous	250	2.1%
Clear cell	392	3.3%
Others	1,548	13.1%
**Grade**		
I	393	3.3%
II	1,368	11.6%
III	6,160	52.3%
IV	3,852	32.7%
**Laterality**		
Unilateral	4,969	42.2%
Bilateral	6,804	57.8%
**Tumor size, mm**		
<80	5,315	45.1%
≥ 80	6,458	54.9%
**T stage**		
T1	343	2.9%
T2	721	6.1%
T3	10,709	91.0%
**N stage**		
N0	7,098	60.3%
N1	4,675	39.7%
**M stage**		
M0	8,521	72.4%
M1	3,252	27.6%
**Surgery**		
No	165	1.4%
Yes	11,608	98.6%
**Radiation therapy**		
No	11,616	98.7%
Yes	157	1.3%
**Chemotherapy**		
No	1,652	14.0%
Yes	10,121	86.0%

### Comparison of Overall and Conditional Survival

**Figure 2:**
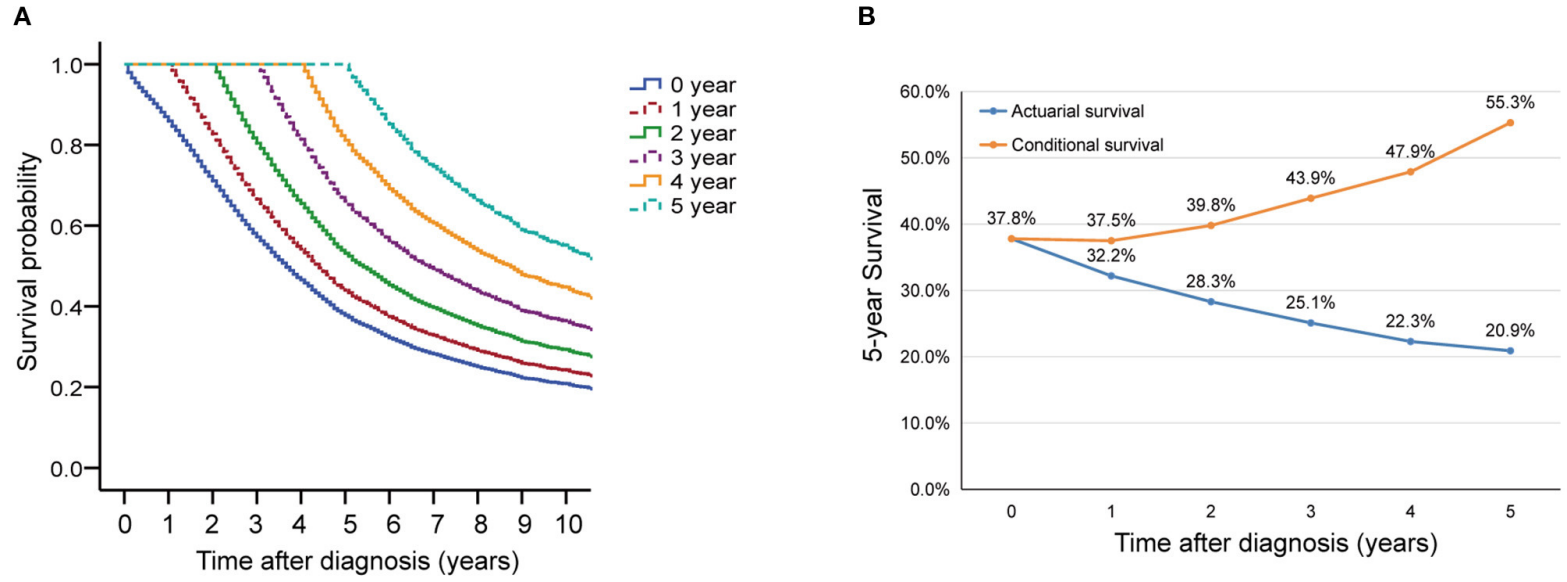
Overall survival (OS) and conditional survival for all patients. **(A)** Kaplan-Meier estimates of survival at diagnosis (0 years) and conditional survival, according to years already survived after diagnosis (1–5 years). **(B)** 5 year conditional survival (CS5) and corresponding OS among paitients with advanced epithelial ovarian cancer.

In this cohort, the 3 and 5 year survival rates were 57.2 and 37.8%, respectively. The median survival was 44 (95% CI, 42.8–45.1) months (Figure 2A). Conditional overall survival probabilities are shown in Table 2, and survival curves in relation to the number of years already survived after diagnosis are shown in Figure 2A. For example, among patients surviving at 1, 2, 3 and 4 years after surgery, the probability of survival at 5 years was 44.0, 53.2, 66.1, and 81.1%, respectively. Then, as summarized in Figure 2B, actuarial OS declined over time, while CS5 continued to increase. The actuarial OS was 32.2% at the sixth year, 28.3% at the seventh year and 25.1% at the eighth year, and the corresponding CS5 was 37.5% at the first year, 39.8% at the second year and 43.9% at the third year.

**Table 2:** Conditional overall survival estimates.

**Total survival** **time (year)**	**If patient has survived (year)**									
	**0**	**1**	**2**	**3**	**4**	**5**	**6**	**7**	**8**	**9**
1	85.9%									
2	71.1%	82.8%								
3	57.2%	66.6%	80.5%							
4	46.6%	54.2%	65.5%	81.5%						
5	37.8%	44.0%	53.2%	66.1%	81.1%					
6	32.2%	37.5%	45.3%	56.3%	69.1%	85.2%				
7	28.3%	32.9%	39.8%	49.5%	60.7%	74.9%	87.9%			
8	25.1%	29.2%	35.3%	43.9%	53.9%	66.4%	78.0%	88.7%		
9	22.3%	26.0%	31.4%	39.0%	47.9%	59.0%	69.3%	78.8%	88.8%	
10	20.9%	24.3%	29.4%	36.5%	44.8%	55.3%	64.9%	73.9%	83.3%	93.7%

### Factors Associated With Overall and Conditional Survival

Upon multivariate analysis at the time of diagnosis, patients who were older, black, unmarried, had mucinous or clear cell histology, had low-grade tumors, had low T and M stages, and underwent radiation therapy had significantly lower OS rates than the controls (all *P* < 0.05, Table 3). Patients with a tumor size ≥ 80 mm who underwent surgery and chemotherapy had significantly higher OS rates (all *P* < 0.05).

**Table 3:** Cox proportional hazards regression analyses of overall survival at the time of diagnosis and conditional survival for advanced EOC survivors at 1, 3, and 5 years after diagnosis.

**Variables**	**At diagnosis**		**1 year after diagnosis**		**3 years after diagnosis**		**5 years after diagnosis**	
	**HR (95% CI)**	***P-*value**	**HR (95% CI)**	***P***	**HR (95% CI)**	***P-*value**	**HR (95% CI)**	***P-*value**
**Age, y**								
<45	Reference		Reference		Reference		Reference	
45–64	1.165 (1.063–1.277)	0.001	1.206 (1.087–1.337)	<0.001	1.148 (0.995–1.326)	0.059	1.088 (0.862–1.372)	0.479
≥65	1.579 (1.433–1.740)	<0.001	1.529 (1.370–1.706)	<0.001	1.439 (1.231–1.681)	<0.001	1.703 (1.324–2.191)	<0.001
**Race**								
White	Reference		Reference		Reference		Reference	
Black	1.207 (1.101–1.324)	<0.001	1.201 (1.077–1.340)	0.001	1.084 (0.908–1.294)	0.375	0.940 (0.678–1.303)	0.711
Other	0.982 (0.900–1.071)	0.675	0.943 (0.853–1.043)	0.252	0.991 (0.855–1.147)	0.901	1.107 (0.873–1.404)	0.403
**Marital status**								
Single	Reference		Reference		Reference		Reference	
Married	0.867 (0.812–0.926)	<0.001	0.914 (0.847–0.986)	0.020	0.957 (0.853–1.073)	0.450	0.935 (0.771–1.135)	0.496
Divorced or Separated	1.005 (0.921–1.096)	0.915	1.051 (0.950–1.162)	0.333	1.018 (0.871–1.189)	0.827	1.085 (0.838–1.405)	0.537
Widowed	1.064 (0.974–1.162)	0.168	1.077 (0.970–1.195)	0.167	1.090 (0.928–1.280)	0.295	1.107 (0.844–1.450)	0.463
**Histological type**								
Serous	Reference		Reference		Reference		Reference	
Endometrioid	0.891 (0.793–1.001)	0.051	0.792 (0.693–0.906)	0.001	0.688 (0.568–0.832)	<0.001	0.770 (0.586–1.012)	0.061
Mucinous	2.407 (2.061–2.811)	<0.001	1.192 (0.923–1.539)	0.178	0.590 (0.368–0.945)	0.028	0.307 (0.126–0.749)	0.009
Clear cell	1.853 (1.629–2.108)	<0.001	1.467 (1.244–1.730)	<0.001	0.996 (0.748–1.326)	0.977	0.621 (0.339–1.139)	0.124
Others	1.351 (1.263–1.445)	<0.001	1.097 (1.010–1.191)	0.029	0.950 (0.835–1.079)	0.428	0.823 (0.661–1.024)	0.081
**Grade**								
I	Reference		Reference		Reference		Reference	
II	1.799 (1.523–2.125)	<0.001	1.767 (1.459–2.141)	<0.001	1.720 (1.324–2.234)	<0.001	1.483 (1.013–2.172)	0.043
III	1.915 (1.634–2.245)	<0.001	1.884 (1.572–2.259)	<0.001	1.784 (1.395–2.281)	<0.001	1.469 (1.030–2.095)	0.034
IV	1.964 (1.671–2.309)	<0.001	1.874 (1.558–2.254)	<0.001	1.921 (1.493–2.471)	<0.001	1.661 (1.148–2.401)	0.007
**Laterality**								
Unilateral	Reference		Reference		Reference		Reference	
Bilateral	1.046 (0.995–1.098)	0.076	1.091 (1.031–1.155)	0.003	1.157 (1.063–1.260)	0.001	1.320 (1.144–1.524)	<0.001
**Tumor size, mm**								
<80	Reference		Reference		Reference		Reference	
≥80	0.845 (0.805–0.886)	<0.001	0.823 (0.779–0.869)	<0.001	0.900 (0.830–0.975)	0.010	0.984 (0.859–1.128)	0.820
**T stage**								
T1	Reference		Reference		Reference		Reference	
T2	1.431 (1.173–1.746)	<0.001	1.360 (1.072–1.725)	0.011	1.101 (0.789–1.536)	0.572	0.874 (0.532–1.435)	0.594
T3	2.178 (1.831–2.590)	<0.001	2.168 (1.764–2.665)	<0.001	1.895 (1.429–2.513)	<0.001	1.401 (0.927–2.117)	0.109
**N stage**								
N0	Reference		Reference		Reference		Reference	
N1	1.010 (0.961–1.061)	0.699	0.998 (0.944–1.056)	0.958	0.977 (0.898–1.063)	0.585	0.880 (0.761–1.107)	0.083
**M stage**								
M0	Reference		Reference		Reference		Reference	
M1	1.506 (1.431–1.584)	<0.001	1.398 (1.318–1.483)	<0.001	1.286 (1.174–1.410)	<0.001	1.137 (0.968–1.335)	0.117
**Surgery**								
No	Reference		Reference		Reference		Reference	
Yes	0.343 (0.290–0.406)	<0.001	0.531 (0.399–0.708)	<0.001	1.076 (0.536–2.163)	0.837	1.323 (0.328–5.340)	0.694
**Radiation therapy**								
No	Reference		Reference		Reference		Reference	
Yes	1.372 (1.140–1.650)	0.001	1.358 (1.093–1.686)	<0.001	1.464 (1.069–2.005)	0.018	1.610 (0.962–2.696)	0.070
**Chemotherapy**								
No	Reference		Reference		Reference		Reference	
Yes	0.679 (0.638–0.724)	<0.001	1.061 (0.974–1.156)	0.177	1.146 (1.009–1.301)	0.035	1.148 (0.938–1.405)	0.182

At 1 year after diagnosis, multivariate analysis showed that age, race, marital status, histological type, tumor grade, size, laterality, T stage, M stage, surgery, radiation therapy, and chemotherapy were independent prognostic factors for patients with advanced EOC (all *P* < 0.05, Table 3). At 3 years after diagnosis, age, histological type, tumor grade, size, laterality, T stage, M stage, radiation therapy, and chemotherapy were independent prognostic factors (all *P* < 0.05, Table 3). Finally, for patients who survived 5 years after diagnosis, only age 65 years or older (HR = 1.703, 95% CI, 1.324–2.191, *P* < 0.001), mucinous histology (HR = 0.307, 95% CI, 0.126–0.749, *P* = 0.009), advanced tumor grade (II: HR = 1.483, 95% CI, 1.013–2.172; III: HR = 1.469, 95% CI, 1.030–2.095; IV: HR = 1.661, 95% CI, 1.148–2.401, all *P* < 0.05), and bilateral tumors (HR = 1.320, 95% CI, 1.144–1.524, *P* < 0.001, Table 3) were persistently significant prognostic factors.

### Subgroup Analysis of Overall and Conditional Survival

The actuarial survival rates and corresponding CS5 for all patients and according to clinical and tumoral characteristics are shown in Tables 4, 5. Overall, CS5 was higher than actuarial survival in each subgroup (Table 4). For patients who survived longer, the difference between the two assessments was generally much larger. For example, the sixth year actuarial survival for patients with M0 was 35.9%, and the CS5 of the first year was 40.5% (**Δ** = 4.6%). The actuarial survival for patients with M0 at the tenth year was 23.7 vs. 56.2% for patients who had already survived for 5 years (**Δ** = 32.5%).

**Table 4:** Actuarial survival rates of patients in relationship to clinical and tumor characteristics.

	**1 year**	**6 year**	**Δ**	**8 year**	**Δ**	**10 year**	**Δ**
**All patients**	85.9%	32.2%	5.3%	25.1%	18.8%	20.9%	34.4%
**Age, y**							
<45	89.4%	44.3%	5.2%	36.2%	17.1%	32.0%	32.9%
45–64	89.3%	35.4%	4.2%	28.5%	17.8%	24.2%	35.1%
≥65	80.5%	25.0%	6.0%	17.8%	18.5%	13.4%	30.1%
**Race**							
White	86.4%	32.5%	5.1%	25.4%	18.6%	21.1%	34.1%
Black	80.8%	24.7%	5.9%	18.5%	20.3%	16.8%	40.7%
Other	85.7%	35.0%	5.8%	27.3%	18.2%	22.5%	32.8%
**Marital status**							
Single	83.9%	33.5%	6.4%	25.6%	19.9%	21.9%	35.4%
Married	88.6%	34.6%	4.5%	27.5%	17.7%	22.8%	33.6%
Divorced or Separated	85.1%	29.7%	5.2%	22.8%	20.5%	18.9%	35.3%
Widowed	77.9%	22.7%	6.4%	16.1%	18.3%	12.9%	31.7%
**Histological type**							
Serous	89.4%	32.6%	3.9%	25.0%	16.7%	20.5%	32.2%
Endometrioid	86.5%	45.6%	7.1%	38.1%	21.4%	32.0%	30.6%
Mucinous	51.5%	24.9%	23.4%	22.5%	47.4%	21.5%	63.1%
Clear cell	73.2%	26.8%	9.0%	21.5%	28.6%	21.5%	53.9%
Others	74.7%	26.9%	9.1%	21.3%	23.9%	18.1%	41.6%
**Grade**							
I	89.3%	53.1%	6.4%	47.2%	17.4%	41.2%	26.6%
II	85.3%	34.9%	6.0%	28.3%	19.6%	23.4%	34.0%
III	85.4%	30.8%	5.3%	23.4%	19.0%	20.0%	35.6%
IV	86.8%	31.0%	4.7%	24.1%	17.4%	18.4%	31.2%
**Laterality**							
Unilateral	83.1%	33.3%	6.8%	26.9%	21.4%	23.3%	38.0%
Bilateral	88.0%	31.5%	4.3%	23.9%	17.1%	19.2%	31.6%
**Tumor size, mm**							
<80	86.4%	28.2%	4.4%	21.6%	18.7%	17.7%	34.7%
≥80	85.6%	35.5%	6.0%	28.0%	18.5%	23.5%	33.5%
**T stage**							
T1	88.0%	56.7%	7.7%	47.8%	17.6%	42.6%	26.7%
T2	84.7%	45.3%	8.2%	39.0%	23.0%	35.3%	36.4%
T3	86.0%	30.7%	5.0%	23.5%	18.2%	19.3%	33.7%
**N stage**							
N0	85.9%	31.0%	5.1%	23.6%	18.0%	19.4%	32.6%
N1	86.0%	34.2%	5.6%	27.6%	20.0%	23.3%	36.9%
**M stage**							
M0	88.6%	35.9%	4.6%	28.1%	17.4%	23.7%	32.5%
M1	79.0%	22.6%	6.0%	17.2%	20.7%	13.6%	37.7%
**Surgery**							
No	41.8%	7.3%	10.2%	6.1%	36.9%	6.1%	67.4%
Yes	86.6%	32.6%	5.0%	25.4%	18.5%	21.1%	34.0%
**Radiation therapy**							
No	86.0%	32.4%	5.3%	25.3%	18.9%	21.0%	34.3%
Yes	79.6%	22.8%	5.8%	14.7%	15.6%	14.7%	36.5%
**Chemotherapy**							
No	65.0%	25.9%	13.9%	21.5%	27.8%	19.5%	44.2%
Yes	89.4%	33.2%	3.9%	25.6%	17.5%	20.9%	32.7%

***Δ**CS5 minus corresponding to actuarial survival*.

**Table 5:** CS5 of patients in different subgroups of clinicopathologic characteristics.

**If patient has survived (year)**,	**0**	**1**	**2**	**3**	**4**	**5**
**All patients**	37.8%	37.5%	39.8%	43.9%	47.9%	55.3%
**Age, y**							
<45	49.3%	49.5%	49.6%	53.3%	56.5%	64.9%
45–64	41.0%	39.6%	41.9%	46.3%	50.6%	59.3%
≥65	30.8%	31.0%	33.3%	36.3%	39.8%	43.5%
**Race**							
White	38.2%	37.6%	39.9%	44.0%	48.0%	55.2%
Black	29.2%	30.6%	35.1%	38.8%	46.2%	57.5%
Other	40.7%	40.8%	41.8%	45.5%	47.5%	55.3%
**Marital status**							
Single	38.2%	39.9%	43.7%	45.5%	49.4%	57.3%
Married	40.4%	39.1%	40.5%	45.2%	49.1%	56.4%
Divorced or Separated	34.9%	34.9%	37.6%	43.3%	45.5%	54.2%
Widowed	28.9%	29.1%	32.3%	34.4%	41.4%	44.6%
**Histological type**							
Serous	38.9%	36.5%	37.8%	41.7%	45.4%	52.7%
Endometrioid	51.1%	52.7%	56.3%	59.5%	57.5%	62.6%
Mucinous	25.4%	48.3%	65.1%	69.9%	77.6%	84.6%
Clear cell	28.5%	35.8%	43.1%	50.1%	62.7%	75.4%
Others	30.3%	36.0%	40.6%	45.2%	51.9%	59.7%
**Grade**							
I	60.8%	59.5%	64.3%	64.6%	69.2%	67.8%
II	40.8%	40.9%	44.6%	47.9%	51.3%	57.4%
III	36.0%	36.1%	38.7%	42.4%	46.8%	55.6%
IV	37.1%	35.7%	35.9%	41.5%	43.5%	49.6%
**Laterality**							
Unilateral	38.0%	40.1%	43.8%	48.3%	52.8%	61.3%
Bilateral	37.8%	35.8%	37.2%	41.0%	44.4%	50.8%
**Tumor size, mm**							
<80	33.8%	32.6%	35.3%	40.3%	45.4%	52.4%
≥80	41.2%	41.5%	43.3%	46.5%	49.8%	57.0%
**T stage**							
T1	61.5%	64.4%	63.9%	65.4%	72.2%	69.3%
T2	49.2%	53.5%	59.1%	62.0%	63.9%	71.7%
T3	36.4%	35.7%	37.6%	41.7%	45.6%	53.0%
**N stage**							
N0	37.3%	36.1%	37.7%	41.6%	45.0%	52.0%
N1	38.7%	39.8%	43.1%	47.6%	52.5%	60.2%
**M stage**							
M0	42.2%	40.5%	42.3%	45.5%	49.6%	56.2%
M1	26.5%	28.6%	31.6%	37.9%	41.8%	51.3%
**Surgery**							
No	8.3%	17.5%	25.8%	43.0%	48.8%	73.5%
Yes	38.3%	37.6%	39.8%	43.9%	48.0%	55.1%
**Radiation therapy**							
No	38.0%	37.7%	39.9%	44.2%	48.1%	55.3%
Yes	28.7%	28.6%	30.0%	30.3%	40.6%	51.2%
**Chemotherapy**							
No	30.6%	39.8%	44.0%	49.3%	55.7%	63.7%
Yes	39.0%	37.1%	39.1%	43.1%	46.5%	53.6%

**Figure 3:**
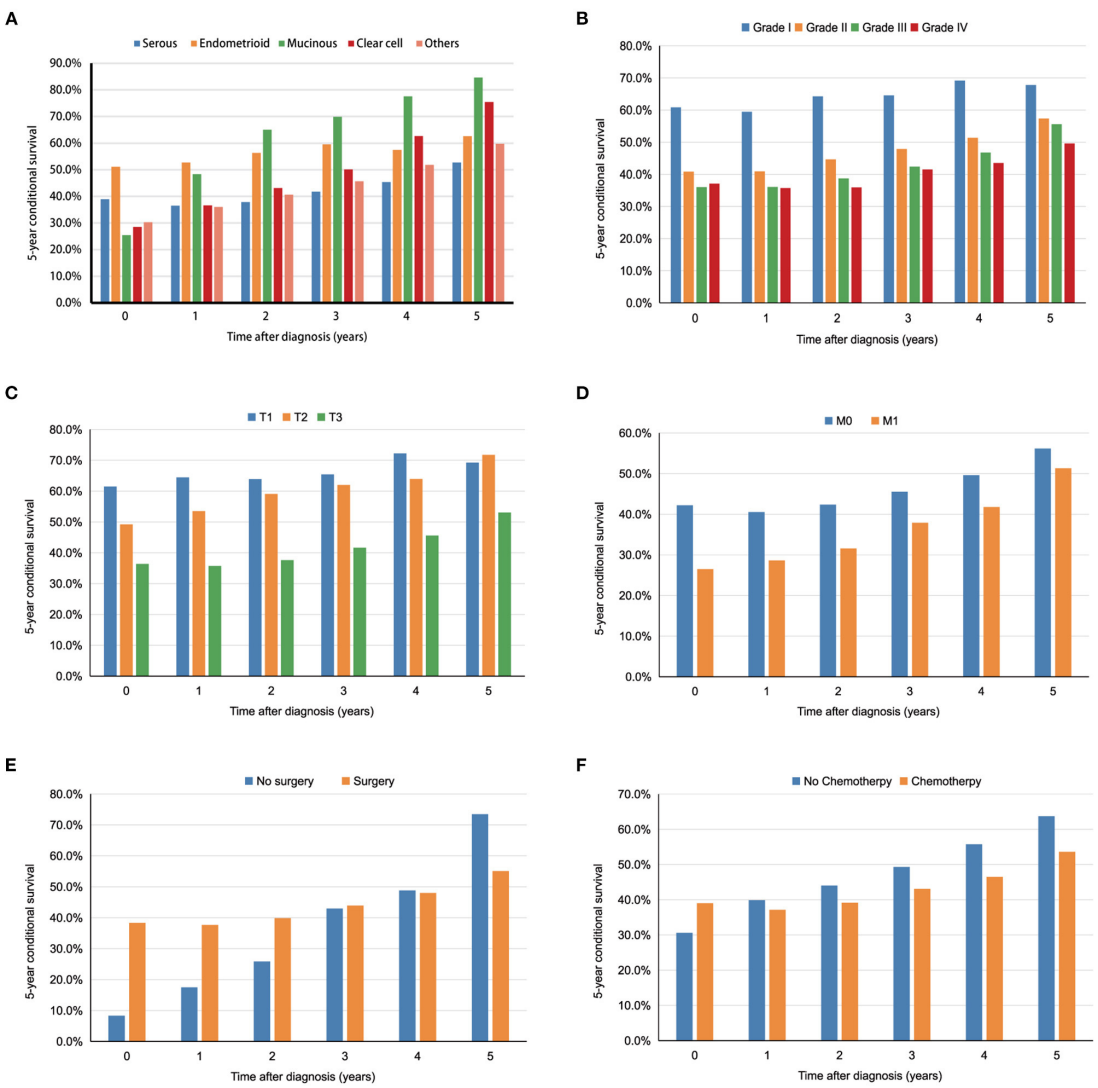
The 5 year conditional survival (CS5) among subgroups divided by histological types **(A)**, tumor grade **(B)**, T stage **(C)**, M stage **(D)**, surgery **(E)**, and chemotherapy **(F)**.

The relationship between the changes in CS5 and pathological prognostic factors (histologic type, grade, T stage and M stage) is shown in Figures 3A–D. Subgroup analysis according to histologic type showed that CS5 was prolonged with increasing survival time in patients with different pathological types (Table 5). Over the first 5 years after diagnosis, CS5 improved from 36.5 to 52.7% in the serous group, 52.7 to 62.6% in the endometrioid group, 48.3 to 84.6% in the mucinous group, 35.8 to 75.4% in the clear cell group, and 36.0 to 59.7% in the other histological type group (Figure 3A). The CS5 of grade I improved from 59.5 to 67.8% after 5 years, while that of grade II/III/IV improved from 40.9/36.1/35.7 to 57.4%/55.6%/49.6% (Figure 3B). Subanalysis according to the T stage showed the same trend, with CS5 of T3 stage increasing from 35.7 to 53.0% after 5 years, while T1/T2 stage increased from 64.4/53.5 to 69.3%/71.7% (Figure 3C). Similarly, CS5 of M1 stage improved from 28.6 to 51.3% after 5 years, while M0 stage improved from 40.5 to 56.2% (Figure 3D). Thus, more dramatic increases were observed in poorer pathological prognostic factors. Furthermore, some subgroup analyses (surgery and chemotherapy) showed that the difference in survival between the two groups reversed over time (Figures 3E,F), which may be because most of the deaths occurring immediately after diagnosis were high-risk patients.

## Discussion

To the best of our knowledge, this is the first study to focus on CS in patients with advanced EOC. In this study, the CS of advanced EOC increased over time, while the actuarial OS declined. The improvements in CS5 rates and the survival gaps between OS and CS were more clear for patients with poor pathological prognostic factors. Furthermore, multivariate analysis confirmed that age, tumor grade and histological type were significant prognostic factors at the time of diagnosis, and their prognostic effects persisted until 5 years after diagnosis. Our results demonstrated that CS analysis could provide more comprehensive prognostic information and help the clinical follow-up of patients with advanced EOC.

EOC is the most common type of ovarian cancer, and approximately 70% of cases are advanced disease (FIGO stage III and IV) at the time of diagnosis (4). However, the prognosis of advanced EOC varies. Traditional survival calculated from the time of diagnosis is less meaningful and may even be misleading for patients who have already survived for a certain amount of time after cancer diagnosis because the prognosis of each individual patient changes over time (7). In this circumstance, conditional survival considers the number of years that a patient has already survived when estimating the survival probability, which could more precisely predict long-term survival on an individual basis (13). In this study, we found that the CS generally increased over time, indicating that the residual risk of death substantially diminished over time. Furthermore, the CS5 was higher than the corresponding OS because the CS5 rate includes the probability of patients who died within a certain number of years after diagnosis. For example, the CS5 at 2 years after diagnosis was 39.8% for all patients with advanced EOC, while the corresponding 7 year OS was 28.3%. Therefore, for patients who survive, the CS rate can offer more accurate information regarding survival estimation for advanced EOC compared with the traditionally used OS rate, as shown in studies of conditional survival in other malignancies (14, 15).

In the present study, we found that older age, black race, mucinous histology, larger tumor size, advanced grade, higher T stage, and M1 stage were independent risk factors for actual OS. However, marriage, surgery and chemotherapy were independent protective factors for advanced EOC, which was consistent with previous studies (16–18). Primary cytoreductive surgery (PCS) followed by chemotherapy has been the standard treatment approach in advanced EOC (19). Surgery can maximally reduce the tumor burden, and chemotherapy kills as many residual cancer cells as possible (16, 20). Wang et al. found that unmarried EOC patients, including divorced/separated, widowed, and single EOC patients, are at greater risk of overall mortality and EOC-specific mortality (17), which is similar to the findings of this study. Multivariate analyses of CS at 1, 3 and 5 years after diagnosis confirmed that only age, histological type, tumor grade and laterality were persistently significant independent prognostic factors even at 5 years after diagnosis, whereas surgery and chemotherapy were not. This finding suggests that patients with high risk might benefit from adjuvant treatments, including systemic therapy, after initial surgery.

Subgroup analysis stratified by the clinicopathological characteristics found that CS was higher than actuarial survival in each subgroup. For patients with poor prognostic factors, such as advanced tumor grade, advanced T stage and M1 stage, the growth trend of CS was significantly better than that of the control group, which was similar to the other tumors (7, 21, 22). For example, the CS5 of M0 patients increased from 40.5% at 1 year to 56.2% at 5 years after diagnosis, while that of M1 patients improved from 28.6 to 51.3%. The reason for this phenomenon may be that some high-risk patients die soon after diagnosis, while the prognoses of surviving patients with high-risk factors will be close to those of patients with some low-risk factors over time; this can also reduce anxiety and improve quality of life, especially for high-risk patients (23). In particular, the subgroup analysis according to surgery and chemotherapy showed that the survival difference between the two groups was reversed over time. This may be because most patients without surgery and chemotherapy died soon after diagnosis, and the remaining surviving patients may have had other protective factors (such as young age and low tumor grade), resulting in better prognosis than the control group. Thus, we should change our conventional concept of treatment and adopt more aggressive strategies to patients survived for certain years.

This study has several limitations. First, this was a retrospective study; thus, there was a certain degree of selection bias. Second, due to the lack of information on tumor dissemination, residual tumors after surgery, the type of chemotherapy and maintenance therapy in the SEER database, the impact of these factors on CS was not further analyzed in this study. Last, because the SEER database lacked information on Asian and European patients, and no other database was included, the universality of our conclusions was reduced. Nonetheless, the SEER database is a well-known national database that provides both a large cohort size and long-term follow-up, two necessary components for studying conditional survival. This study is the first to focus on CS of advanced EOC, which could support dynamic prognosis assessment and enable accurate and individualized follow-up strategies.

## Conclusion

In conclusion, CS estimates of advanced EOC generally increase over time, especially for patients with poor pathological factors at baseline. Age, histological type, tumor grade and laterality remained significant prognostic factors even 5 years after diagnosis. Our study provides an effective way to dynamically evaluate the long-term survival of advanced EOC and recommends the need for continuing surveillance and care in long-term survivors.

## Data Availability Statement

The original contributions presented in the study are included in the article/supplementary material, further inquiries can be directed to the corresponding author/s.

## Ethics Statement

All authors have signed the SEER Research Data Agreement to protect the privacy of patients, which is consistent with ethical principles.

## Author Contributions

PeZ and GC designed the experiments and wrote the manuscript. PiZ collected the data. PeZ and PiZ contributed to the statistical analysis of the data. All authors read and approved the final manuscript.

## Conflict of Interest

The authors declare that the research was conducted in the absence of any commercial or financial relationships that could be construed as a potential conflict of interest.

## Publisher's Note

All claims expressed in this article are solely those of the authors and do not necessarily represent those of their affiliated organizations, or those of the publisher, the editors and the reviewers. Any product that may be evaluated in this article, or claim that may be made by its manufacturer, is not guaranteed or endorsed by the publisher.
